# The trough of disillusionment: A critique of the “transition” paradigm

**DOI:** 10.1192/j.eurpsy.2021.68

**Published:** 2021-08-13

**Authors:** S. Gülöksüz

**Affiliations:** Psychiatry And Neuropsychology, Maastricht University, Maastricht, Netherlands

**Keywords:** psychosis, early intervention, public health, clinical high-risk

## Abstract

I will attempt to address the issues surrounding the CHR concept in light of novel data and briefly discuss emerging alternatives. The root problem of the CHR early invention strategy is the exertion of reducing early nonspecific (pluripotent) psychopathology to a unidimensional model restricted only to positive psychotic symptoms, which define the binary categories of CHR and “transition” in help-seeking populations. This major conceptual handicap undermines the validity and clinical utility. The core predictor of the “transition” rate is the degree of the risk-enrichment and not the CHR status. Even with a significant pretest risk enrichment, the prognostic accuracy is mediocre. The incidence and “transition” rates of CHR in the community are very low; therefore, CHR does not represent a cost-effective clinical target—prevention paradox. CHR succeeding early pluripotent psychopathology is already late for intervention. “Transition” is not a categorical progression but a unidimensional shift in psychotic symptoms, and therefore, influenced by the fluctuation of psychotic symptoms, leading to both false positives and underestimation of nonpsychotic psychopathology. There exists no evidence for a specific effect of any intervention in preventing “transition”; therefore, CHR is not an ideal treatment target. Binary “transition” outcome does not represent a valid phenotype for research as “transition” rates are primarily driven by the sampling heterogeneity. The multidimensional psychopathology and functioning are more clinically relevant, overarching, and service-user-centered measures to define individual risk and outcome. Guided by the public health perspective, a universal early intervention framework, underscoring improved access to care, may represent a better strategy.

**Disclosure:**

No significant relationships.

